# Comprehensive update on the monkeypox outbreak

**DOI:** 10.3389/fmicb.2022.1037583

**Published:** 2022-11-10

**Authors:** Asad Mustafa Karim, Jeong Eun Kwon, Mujahid Aizaz Karim, Haseeb Iftikhar, Muhammad Yasir, Irfan Ullah, Se Chan Kang

**Affiliations:** ^1^Department of Oriental Medicine Biotechnology, College of Life Science, Kyung Hee University, Yongin, Gyeonggi, South Korea; ^2^Sheikh Zayed Hospital and Medical College, Rahim Yar Khan, Punjab, Pakistan; ^3^Special Infectious Agents Unit, King Fahd Medical Research Center, King Abdulaziz University, Jeddah, Saudi Arabia; ^4^Department of Medical Laboratory Sciences, Faculty of Applied Medical Sciences, King Abdulaziz University, Jeddah, Saudi Arabia; ^5^Department of Internal Medicine, Section of Infectious Diseases, Yale University School of Medicine, New Haven, CT, United States

**Keywords:** monkeypox virus, monkeypox vaccine, monkeypox outbreak, tecovirimat, orthopoxvirus infections

## Abstract

Monkeypox (MPX) was first reported in 1970 in humans and outbreaks were restricted and highly localised to endemic regions of western and central Africa. However, after the first reported case in the UK in early May, 2022, the pattern of epidemic spreading in the geographical regions was much larger compared to past, posing a risk MPX might become entrenched beyond endemic areas. This virus is less transmissible than SARS-CoV-2, as it transmitted mainly through personal, close, often skin-to-skin contact with infectious MPX rash, body fluids, or scabs from an individual with MPX. Infections usually present with chills, fever, fatigue, muscle aches, headache, sore throat, skin lesions, and lymphadenopathy. Currently, there are no antivirals approved for MPX. However, an antiviral drug called “tecovirimat,” approved for the treatment of smallpox, has been made accessible to treat MPX. Moreover, to prevent MPX, there are two vaccines available which are approved by FDA: Bavarian Nordic JYNNEOS, and ACAM2000 vaccine. Contact tracing is absent in case of MPX outbreak and there is lack of information from the data systems in rapid manner. Additionally, test capacity needs to be increased. Like SARS-CoV-2, global MPX outbreak demand for vaccines far exceeds availability.

## Introduction

**Figure 1:**
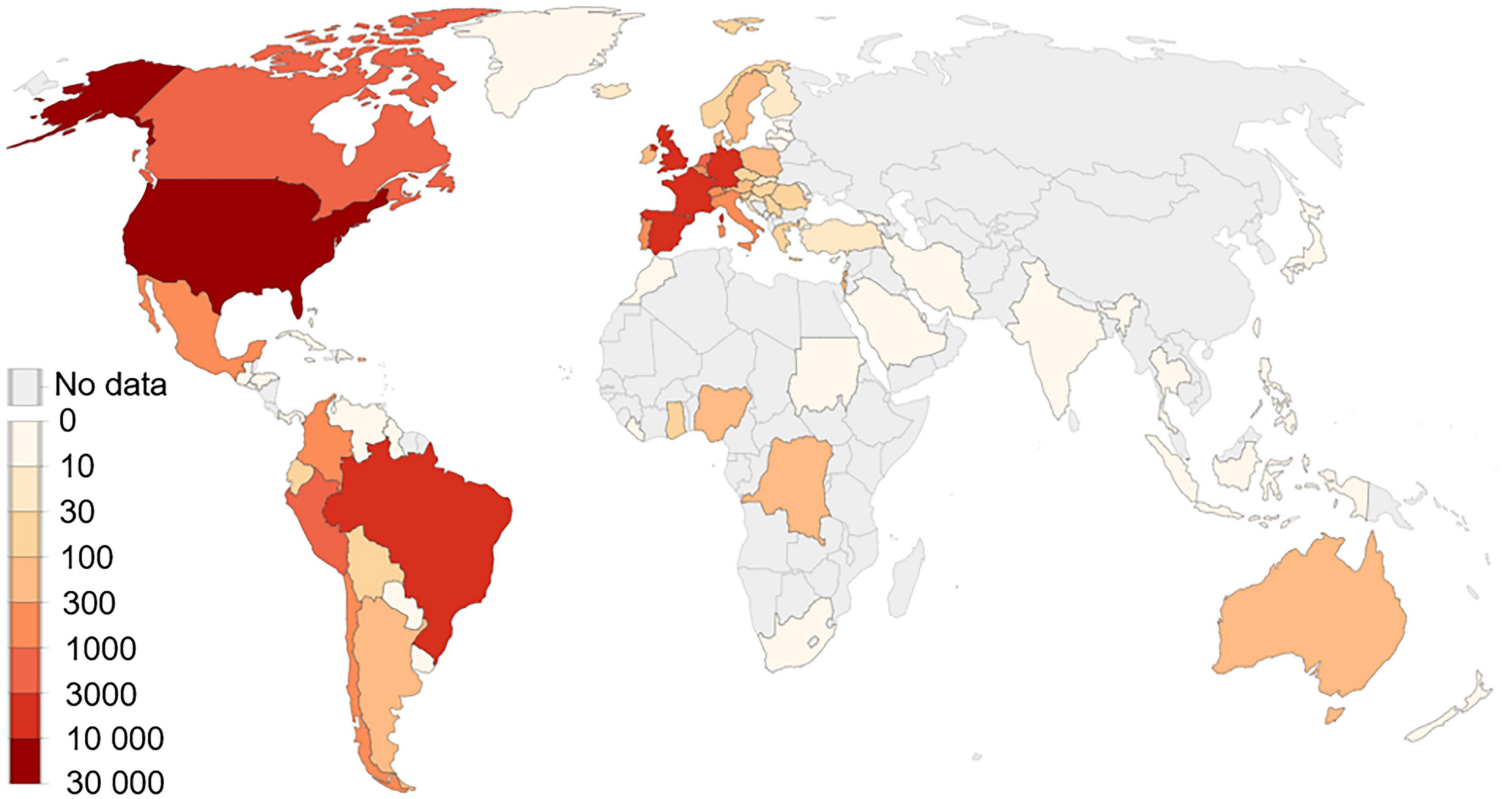
Global map of cumulative confirmed cases in endemic and non-endemic countries.

Human monkeypox virus (MPXV) is spreading in the USA and Europe among people who have not travelled to endemic areas. On July 23, 2022, monkeypox (MPX) outbreak was declared a public health emergency of international concern by WHO and called for a coordinated international response to slow the spread of disease (Nuzzo et al., 2022). It is the second time in 2 years that WHO has taken this step, ranking MPXV amongst 2 other diseases, polio and COVID-19, that currently carry the classification (Nuzzo et al., 2022). MPX is caused by virus transmitted between animals and humans and from humans to humans by close contact with lesions, body fluids, respiratory droplets and contaminated materials (Guarner et al., 2022). As of September 6, 2022, there have been 42,516 confirmed cases from 93 nonendemic countries which have not historically reported MPX (Figure 1; CDC, 2022a). MPXV was first reported in 1970 in humans and outbreaks were restricted and highly localised to endemic regions of western and central Africa. In 2003, the first monkeypox outbreak was reported in United States but it linked to animals imported from Ghana. However, after the first reported case in the UK in early May, 2022, the pattern of epidemic spreading in the geographical regions was much larger compared to past, posing a risk MPXV might become entrenched beyond endemic areas. Since May, the highest number of cases have been reported in nonendemic countries like US (*n* = 19,996), Spain (*n* = 6,543), Germany (*n* = 3,493), Brazil (5037), and the UK (*n* = 3,413) and these clusters represent “an extraordinary event” (Figure 1; CDC, 2022a). However, these reported cases are likely to be an underestimation of the actual numbers because of inadequate clinical recognition of MPXV infection and the long incubation period of the virus (5–21 days; WHO, 2022). Given the rapid pace with which cases are being diagnosed, a coordinated international response is essential.

## Monkeypox transmission

As MPX cases continue to soar, scientists are learning more about the spread of disease. Early claims that the virus spreads mainly through repeated skin-to-skin contact among individuals have largely borne out, according to a tranche of new studies (Guarner et al., 2022). The pattern of spread and symptoms do not look like what we had observed in endemic West and Central Africa. Though some children and women have been infected, men who have sex with men (MSM) have affected the most so far. The more the MPXV continues to transmit, the more threats it will pose to infect other populations, including wild animals, consequently, establishing viral reservoirs that might infect humans repetitively.

There are two genetic clades of this virus that include the West African clade and the Central African clade. Human-to-human transmission is not documented in case of West African clade and it has a case fatality rate of less than 1%. In case of Central African clade, human-to-human transmission has been reported with a case fatality rate of up to 10% (Moyo et al., 2022). West African clade may cause substantial sickness and it is typically self-limiting. Children are more susceptible for in case of Central African clade. MPXV exposure during pregnancy may result in difficulties, congenital monkeypox, or stillbirth (Moyo et al., 2022). The effect of MPXV disease on pregnancy outcomes with vertical transmission of viral infection to the fetus has also been reported (Mbala et al., 2017).

MPXV is less transmissible than SARS-CoV-2, as it transmitted mainly through personal, close, often skin-to-skin contact with infectious MPX rash, body fluids, or scabs from an individual with MPX (Guarner et al., 2022; Lapa et al., 2022). Direct contact can happen during intimate contact including; oral, anal, vaginal sex or touching the genitals (labia, vagina, penis, and testicles) or anus of a person infected with MPXV (Lapa et al., 2022). It also spreads by massage, hugging, kissing, touching clothing, towels, bedding, and fetish gear of the infected individual (CDC, 2022a). MPXV is not known to linger in the air like SARS-CoV-2. In the existing outbreak, most cases have happened among MSM (CDC, 2022a). MPXV can spread through sexual activity, however it is not considered to be a sexually transmitted disease as it can be acquired without having sex. Furthermore, although the MPXV DNA has been detected in saliva, semen, and other clinical samples, it is yet unclear if MPX can spread through vaginal secretions, semen, urine, or feces (Lapa et al., 2022). In a study by J Tarín-Vicent et al., it was reported that having a larger number of lesions in and around the anus was associated with anal-receptive sex while having more number of lesions in mouth and throat was linked to oral sex (Tarín-Vicente et al., 2022). Given all these findings, the virus transmission at this point is not as simple as reported in earlier monkeypox outbreaks. Moreover, the spread of MPXV can occur across the placenta to the fetus in pregnant women (Tarín-Vicente et al., 2022). In a recent report, human-to-dog transmission of MPXV has been confirmed, suggesting a real canine disease (Seang et al., 2022). Therefore, it is imperative for the scientific community to investigate physical, serological, PCR-based and epidemiological assessments of both symptomatic and close contacts to answer what percentage of infections are asymptomatic; what body sites are infectious; and importance of condoms in sexually active communities.

## Clinical characteristics

MPX has a long incubation period ranging from 5 days to 3 weeks (WHO, 2022). Infections usually present with chills, fever, fatigue, muscle aches, headache, sore throat, skin lesions, and lymphadenopathy (CDC, 2022a). The lesions develop from macules and papules to vesicles and pustules that ulcerate and crust before healing over several weeks (CDC, 2022b). The skin lesions usually happen in crops (CDC, 2022b). The primary rash and lesions are generally located at the site of inoculation (on or near the anus or genitalia). MPX is a self-limited infection and typically lasts for 2–4 weeks.

In addition, recent studies have also shown the presence of virus in asymptomatic individuals with history of contact with known cases (WHO, 2022). Recently, two monkeypox cases have been reported in infants in the USA most likely *via* household transmission. However, most of the cases have occurred among MSM (CDC, 2022a). Moreover, nearly 13 infections have been described among females, including one pregnant women, and at least 2 children have become infected. The mean incubation period from time of exposure to first symptom onset has been estimated at 7.6 days, with 95% of individuals developing symptoms within 17.1 days (CDC, 2022a). The symptoms usually start with all-over feeling of being ill. The characteristic features of MPXV infection include fever, lethargy, chills, headaches, asthenia, myalgia, backaches, and lymph node swellings. After the appearance of prodromal rash, lesions start as macules that grow to papules, vesicles, and then pustules and resolves with crust and scab formation, which spontaneously exfoliate on recovery (CDC, 2022a). Penile edema, anorectal pain, proctitis with bleeding, and balanitis and phimosis are also common symptoms in the present MPX outbreak (Tarín-Vicente et al., 2022). Moreover, epiglottitis, odynophagia sore throat, and tonsillitis also have been reported in patients. According to a study, the most common locations of lesions were anogenital region (73%), trunk, legs, or arms (55%), face (25%), and palms and soles (10%) [9]. Encephalitis, pneumonia, and eye infections are other known complications of MPX, which occur in children, immunocompromised individuals, and pregnant women (Thornhill et al., 2022). Hospitalization is rare but patients with anorectal or oral pain require pain management in hospitals. Individuals infected with MPXV should quarantined or advised to self-isolate themselves for 2–4 weeks. Inflammation of the pharyngeal, genital mucosa, conjunctival are additional symptoms of MPX (Tarín-Vicente et al., 2022).

## Diagnostics and therapeutics

The distinction between monkeypox, chickenpox, and herpesvirus disease remains clinically challenging during outbreaks. The lesions of the MPX vary from those in chickenpox in their extent of skin invasion, and distribution which is very important with MPX lesions. Additionally, in case of chickenpox, the lesions appear denser on the trunk and do not spread throughout the face and extremities as in case of MPX. A clinically distinguishing symptom of MPX infection is lymphadenopathy. Any unfamiliar skin lesion, specifically in the anogenital region, should be examined. The diagnosis of MPXV is based on clinical symptoms and examinations. These include viral culture of anogenital lesion swabs, nasopharyngeal or oropharyngeal swabs and laboratory analysis of skin specimens and exudates from the lesions. Moreover, polymerase chain reaction (PCR) assays using samples from skin lesions (fluid from vesicles and pustules) can be employed for detection. However, blood PCR is not recommended because of its limitations of short duration of viremia. In US, during the current monkeypox outbreak, PCR testing is being used in commercial as well as public health laboratories. Testing should be considered in patients with clinically compatible lesions and an epidemiologic risk factor as well as any patient with a characteristic lesion (deep-seated vesicle or pustule with central umbilication). Non-variola Orthopoxvirus real-time PCR test cleared by CDC’s FDA can detect MPXV and is being used in during the current outbreak. Unfortunately, no data on the use of other sample types such as saliva, blood, or genital secretions for testing is available.

MPXV belongs to *Orthopoxvirus* genus and another member of this genus called as variola virus, causes smallpox. Currently, there are no antivirals approved for MPX. However, an antiviral drug called “tecovirimat,” also known as ST-246 or TPOXX approved for the treatment of smallpox, has been made accessible to treat MPXV through an expanded access Investigational New Drug (IND) protocol (CDC, 2022c; Almehmadi et al., 2022). Tecovirimat is accessible as an injectable drug for intravenous administration and as an oral capsule (Almehmadi et al., 2022). Currently, tecovirimat is only suggested for patients with severe MPX disease or who are at higher risk for severe MPX (immunocompromised people, pregnant or lactating women, or children with atopic dermatitis) and those with concurrent illnesses (CDC, 2022c). Mechanism of action of Tecovirimat includes targeting and inhibition of the activity of the orthopoxvirus VP37 protein (encoded by and highly conserved in all members of the orthopoxvirus genus) and blocking its interaction with cellular Rab9 GTPase and TIP47, which prevents the formation of egress competent enveloped virions necessary for cell to cell and long-range dissemination of virus. Furthermore, numerous clinical trials such as PLATINUM PALM-007, ACTG5418, WHO/ARNS) are underway to provide required data on the efficacy and safety tecovirimat for MPX (CDC, 2022c). For maximum absorption of oral drug, simultaneous intake of a high-fat meal, ideally about 600 calories and 25 g of fat is required (CDC, 2022c). Other possible antiviral agents (such as cidofovir and brincidofovir) could be treatment options in life-threatening cases. However, these antivirals should be used under an IND protocol available from the CDC (CDC, 2022c).

## Vaccines

To prevent MPX, there are two vaccines available which are approved by FDA: Bavarian Nordic JYNNEOS vaccine, and ACAM2000 (CDC, 2022d). ACAM2000 was permitted only for smallpox however, it was granted an expanded-access to use it against MPX. In case of JYNNEOS vaccine, an attenuated, live vaccinia virus, incapable of replicating, is administered as a 2-dose series with high antibody response in 2 weeks after the second dose of vaccine (CDC, 2022d). While ACAM2000 vaccine uses a live vaccinia virus capable of replicating and is administered as a single dose but requires multiple skin punctures (CDC, 2022d). Both vaccines are 85% effective in preventing MPX and can be administered as post exposure prophylaxis.

Among these two vaccines, administration of ACAM2000 has higher risk of adverse events in immunocompromised or people with eczema. ACAM2000 vaccination also has side effects such as myocarditis, lymphadenitis, pericarditis and constitutional symptoms, such as fatigue, malaise, myalgia, fever, and headache. ACAM2000 generates cellular type of immunity in vaccinated individuals. According to a study, in a Phase I clinical trial, ACAM2000 vaccination has been reported to induce positive responses in cell-mediated immunity in 100% of subjects. Hence, post-exposure vaccination can be effective both for preventing the disease and reducing the disease severity. It also has some operational issues such as its administration involves a bifurcated needle (CDC, 2022d). However, JYNNEOS is a better choice. Currently, because of limited supplies of JYNNEOS, health departments in US and Europe are only administering a single dose of vaccine by intradermal injection to high risk people for MPX aged 18 years or older (CDC, 2022d). In near future, it is expected that the total number of doses available for use may increase up to 5-fold because the lower dose is immunologically noninferior to the standard dose, but with more reactogenicity.

## Preventing the spread of MPXV

Appropriate and consistent use of personal protective equipment (PPE) when examining MPX patient is highly protective and prevents transmission to healthcare personnel (HCP; CDC, 2022e). According to the recommendations by CDC, HCP who enter the patient’s room should use gloves, gown, eye protection (i.e., goggles or a face shield that covers the front and sides of the face), and a NIOSH-approved particulate respirator equipped with N95 filters or higher (CDC, 2022e). Confirmed or suspected patients should wear mask and should be placed in a single-person room (CDC, 2022e). Lesions should be covered with a gown or sheet. Individuals with MPX infection should avoid sexual or close contact with others until the rash and lesions are absolutely healed (CDC, 2022e). There are no data available about protection from subsequent MPX infection but because of limited supply of vaccine, persons previously infected should not be prioritized for vaccination.

## Lessons from current outbreaks

After the WHO recommendation, there is growing concern that the MPXV can find an animal reservoir outside Africa. MPX outbreak shows why global health cannot be overlooked. Thus, there is a dire need for global collaboration between public health and veterinary authorities working from a ‘One Health’ perspective. It is very important to manage exposed animals especially pets to prevent the MPXV from being spread to wildlife. MPXV can infect a wide range of mammal species, including anteaters, hedgehogs, prairie dogs, squirrels, shrews and dogs and these animals can be natural hosts and can transmit the disease to humans. Although MPX is endemic in different parts of Africa for decades, however, there was no progress on vaccine development and clinical trials on treatments. After the global MPX outbreak, there is much more to learn. Contact tracing is absent in case of MPX outbreak and there is lack of information from the data systems in rapid manner. Additionally, test capacity needs to be increased. Many persons who are at risk of MPX may not be involved with the health care system, making diagnosis, containment, and prevention challenging. Like SARS-CoV-2, global MPX outbreak demand for vaccines far exceeds availability. Though the supply of tecovirimat has increased, this drug must still be used under an IND protocol, which limits access.

## Discussion

MPX and smallpox vaccines are formulated based on a vaccinia virus and give cross-protection due to immune response to *orthopoxviruses*. Occurrence of current MPX outbreak is likely due to abandonment of the global smallpox vaccination programs, rendering a massive proportion of the world population vulnerable to monkeypox because of loss of immunity against *orthopoxviruses*. Thus, clinical, public-health, and vaccination strategies against members of *orthopoxviruses* should be revisited and reinvigorated.

## Data availability statement

The raw data supporting the conclusions of this article will be made available by the authors, without undue reservation.

## Author contributions

AMK, SCK, and MY: conceived and planned this study. SCK and JEK: supervision, investigation, and resources. AMK, MAK, HI, IU, MY, and JEK: writing – original draft and writing – review and editing.

## Funding

This research was supported by the Ministry of Trade, Industry & Energy (MOTIE), Korea Institute for Advancement of Technology (KIAT).

## Conflict of interest

The authors declare that the research was conducted in the absence of any commercial or financial relationships that could be construed as a potential conflict of interest.

## Publisher’s note

All claims expressed in this article are solely those of the authors and do not necessarily represent those of their affiliated organizations, or those of the publisher, the editors and the reviewers. Any product that may be evaluated in this article, or claim that may be made by its manufacturer, is not guaranteed or endorsed by the publisher.
